# Network pharmacology- and molecular docking-based approaches to unveil the pharmacological mechanisms of dihydroartemisinin against esophageal carcinoma

**DOI:** 10.3389/fgene.2022.1017520

**Published:** 2022-11-25

**Authors:** Haixia Wang

**Affiliations:** Pharmacy Department, West Hospital of the Second Hospital of Shanxi Medical University, Taiyuan, China

**Keywords:** dihydroartemisinin, esophageal carcinoma, network pharmacology, molecular docking, ADORA2B, AURKA

## Abstract

**Objective:** Dihydroartemisinin (DHA) is an active metabolite of artemisinin and its derivatives, which is a potent drug extensively applied in clinical treatment of malaria. The antitumor properties of DHA have received increasing attention. However, there is no systematic summary on the pharmacological mechanisms of DHA against esophageal carcinoma (ESCA). The present study implemented network pharmacology- and molecular docking-based approaches to unveil the pharmacological mechanisms of DHA against ESCA.

**Methods:** DHA targets were accessed through integrating the SwissTargetPrediction, HERB, as well as BATMAN-TCM platforms. In TCGA-ESCA dataset, genes with differential expression were screened between 161 ESCA and 11 normal tissue specimens. DHA targets against ESCA were obtained through intersection. Their biological significance was evaluated with functional enrichment analysis. A prognostic signature was established *via* uni- and multivariate cox regression analyses. DHA-target interactions were predicted *via* molecular docking. Molecular dynamics simulation was implemented to examine the stability of DHA binding to potential targets. **Results:** The study predicted 160 DHA targets as well as 821 genes with differential expression in ESCA. Afterwards, 16 DHA targets against ESCA were obtained, which remarkably correlated to cell cycle progression. The ADORA2B- and AURKA-based prognostic signature exhibited the reliability and independency in survival prediction. The stable docking of DHA-ADORA2B and DHA-AURKA was confirmed.

**Conclusion:** Collectively, this study systematically revealed the basis and mechanism of DHA against ESCA through targeting multi-target and multi-pathway mechanisms, and thus offered theoretical and scientific basis for the clinical application of DHA.

## Introduction

Esophageal carcinoma (ESCA) remains one of the most lethal cancers across the globe (Sung et al., 2021), with two primary histological subtypes: squamous cell carcinoma (ESCC) as well as adenocarcinoma (EAC) (Lu et al., 2022). The overall 5-year survival rate is merely 15–25% (Hulshof et al., 2021). The features and etiology of ESCA may vary on the basis of region or ethnicity (Liu et al., 2017). ESCC occupies the predominant subtype of ESCA, notably in the Asian and African regions, which correlates to dietary habits as well as exposure to carcinogens (Zhang et al., 2022). Oppositely, EAC mainly develops from esophageal epithelial intestinal metaplasia caused by chronic gastroesophageal reflux diseases, which is the majority of ESCA in Western countries (Zhang et al., 2022). Currently, endoscopic or surgical resection is appropriate for early-stage patients, while radio- or/and chemotherapies are mainly reserved for those with advanced stage or metastases (Mukherjee et al., 2021; Yang et al., 2022). Chemotherapeutic agents (cisplatin, 5-fluorouracil, doxorubicin, etc.) may cause a range of dose-limiting toxicities (Zhang et al., 2021). Molecular targeted therapy has achieved unprecedented progress in cancer therapy (Lu et al., 2022). Nevertheless, only trastuzumab against HER2, as well as ramucirumab targeting VEGFR-2 have been recommended for the treatment of ESCA (Xu et al., 2021; Safran et al., 2022). Immunotherapy comprising immune checkpoint inhibitors, vaccines, monoclonal antibodies as well as adoptive cellular immunotherapy represents a novel therapeutic option of ESCA (Janjigian et al., 2021; Shitara et al., 2021). Despite the remarkable potential of immunotherapy in cancer therapy, individual patients’ response greatly varies, and only a small percentage of cases respond to immunotherapy (Luo et al., 2021; Sun et al., 2021). Hence, achievement of the goal of effective therapeutic options remains challenging. Rather than exploring a single ESCA-causing gene and therapeutic agents that act only on a single target, the entire drug-disease network should be considered, aiming to determine multi-target agents to lower side effects.

Traditional Chinese medicine artemisinin is extracted from Artemisia annua L, and dihydroartemisinin (DHA) with high water solubility and antimalarial activity is the first-generation derivative of this compound that is an effective and fast-acting antimalarial agent with low toxicity (Zhu et al., 2020). DHA displays the considerable potential for preventing or treating ESCA because of its low toxicity and known safety, and preclinical research has proposed the molecular mechanisms and pharmacological effect underlying DHA against ESCA (Zhu et al., 2020). Zhao et al. reported that DHA exhibits an anti-proliferative effect against ESCC cells, which is capable of down-regulating mTOR cascade pathway partly *via* binding to AKT1 and p70S6K (Zhu et al., 2020). Pyroptosis is a novel form of pro-inflammatory programmed cell death, and its inducers enable to strengthen antitumor effects (Niu et al., 2022). Song et al. proposed that DHA may trigger pyroptosis of ESCC cells *via* impacting the activity of PKM2-caspase-8/3-GSDME signaling (Jiang et al., 2021). Administration of chemotherapeutic agents is usually accompanied by resistance. DHA may sensitize ESCC to cisplatin through attenuating the activity of Sonic Hedgehog pathway (Cui et al., 2020). There is an interplay between autophagy and epithelial-mesenchymal transition during tumor progression (Chen et al., 2021; Niu et al., 2021). He at al. found that the migratory capacity and the epithelial-mesenchymal transition process of ESCA cells is mitigated by DHA *via* triggering autophagy (Chen et al., 2020). PKM2 is a key regulator of glycolysis, and DHA lowers glycolysis of ESCA through down-regulating PKM2 (Li et al., 2019). Additionally, hTERT has been confirmed as a therapeutic target of DHA against ESCC (Li et al., 2021). Altogether, DHA exerts an anti-ESCA effect through targeting multi-target and multi-pathway mechanisms.

In the current study, we developed a new integrated strategy to probe out the key targets and mechanisms of DHA in the treatment of ESCA on the basis of network pharmacology and molecular docking, which offered the theoretical and scientific basis for the clinical application of DHA against ESCA.

## Materials and methods

### Dihydroartemisinin target prediction

The chemical structure of DHA was acquired from the PubChem chemical information resource (https://pubchem.ncbi.nlm.nih.gov) (Kim et al., 2021). Potential targets of DHA were searched from the SwissTargetPrediction (http://www.swisstargetprediction.ch) (Daina et al., 2019), as well as HERB (http://herb.ac.cn/) (Fang et al., 2021) web servers. Additionally, DHA targets were screened through the BATMAN-TCM (http://bionet.ncpsb.org/batman-tcm) platform in accordance with the screening conditions of score cutoff = 5 and adjusted *p*-value < 0.05 (Liu et al., 2016). DHA targets were matched and corrected on the basis of the universal Protein Resource (UniProt, (2021); http://sparql.uniprot.org/). Afterwards, DHA targets obtained from three platforms were merged, and deduplicated.

### The cancer genome atlas (TCGA)-esophageal carcinoma data acquisition

RNA transcriptome profiles as well as clinicopathological and survival information of ESCA cases were downloaded from TCGA-ESCA dataset (https://cancergenome.nih.gov/). In total, 161 ESCA and 11 normal tissue specimens were enrolled for subsequent analysis.

### Differential expression analysis

In TCGA-ESCA dataset, limma package was adopted for screening differentially expressed genes (DEGs) between ESCA and normal tissue specimens. Adjusted *p*-value < 0.01 together with |log2 fold-change|>1 were set as the screening criteria. Shared genes between DHA targets and DEGs were intersected through the Venny 2.1 online tool, named DHA targets against ESCA.

### Protein-protein interaction (PPI) analysis

DHA targets against ESCA were uploaded to the Search Tool for the Retrieval of Interacting Genes (STRING) database (https://string-db.org/) (Szklarczyk et al., 2015). In accordance with hide unconnected targets along with highest confidence of 0.900, PPI network of DHA targets against ESCA was obtained. Hub genes among DHA targets against ESCA were further evaluated with cytoHubba plugin of Cytoscape tool 3.7.2 (Chin et al., 2014). Degree of each hub gene was also computed.

### Establishment and verification of a prognostic signature

In TCGA-ESCA dataset, univariate cox regression analysis was implemented to screen which DHA targets against ESCA significantly correlated to ESCA cases’ overall survival (OS). Prognostic DHA targets against ESCA with *p*-value <0.05 were enrolled in a multivariate cox regression model. The prognostic signature was conducted on the basis of the linear combination of regression coefficients derived from the multivariate Cox regression model multiplied with expression values. The risk score of each case was computed. TCGA-ESCA cases were equally separated into discovery set as well as validation set. In each set, ESCA cases were further assigned to high- and low-risk subpopulations. Kaplan-Meier curves of OS between two subpopulations were plotted, and OS difference was estimated with log-rank test. Alive and dead status of cases in each set was displayed. Receiver operating characteristic (ROC) curve was drawn for evaluation of the prediction accuracy of the prognostic signature. Area under the curve (AUC) was also computed. TNMplot.com web tool was employed for comparison of survival difference between high and low expression subpopulations of DHA targets against ESCA in TCGA-ESCA dataset (Bartha and Győrffy, 2021). Expression values of DHA targets against ESCA across distinct pathological stages were displayed with ggpubr package, and the difference was evaluated using one-way analysis of variance.

### Uni- and multivariate cox regression analysis

Clinicopathological factors comprising gender, pathological stage, TNM staging as well as prognostic signature were enrolled for estimating their associations with TCGA-ESCA cases’ OS by use of uni- and multivariate cox regression analysis.

### Functional enrichment analysis

Biological features of DHA targets against ESCA were probed out utilizing clusterProfiler package (Yu et al., 2012). Gene Ontology (GO) covering biological process, cellular component and molecular function, and Kyoto Encyclopedia of Genes and Genomes (KEGG) enrichment analyses were separately implemented.

### Gene set enrichment analysis (GSEA)

The potentially altered biological processes between high and low expression subpopulations of DHA targets against ESCA were determined with GSEA tool (Subramanian et al., 2005). The “c5. go.v7.5.1. symbols” and “c2. cp.kegg.v7.5.1. symbols” gene sets acquired from the Molecular Signatures Database Molecular Signatures Database (Liberzon et al., 2015) were utilized as reference sets.

### Molecular docking

The 3D structures of DHA targets against ESCA were accessed from the PDB database and saved in pdb format. PyMOL software was used for protein pretreatment. In addition, the 3D structure of DHA was created using Chem3D software and saved in mol*2 format. The pre-processed targets and DHA were imported into AutoDockTools 1.5.6 software for molecular docking, and the results were saved in pdbqt format. Vina scripts were run for computing molecular binding energies as well as visualizing molecular docking results. Additionally, Discovery Studio 2019 software was implemented for searching for docking sites as well as calculating LibDockScore for flexible binding. The molecular docking results were imported into PyMOL software for visualizing the molecular docking conformation.

### Molecular dynamics simulation

Molecular dynamics simulation was implemented by use of Discovery Studio 2019 software. The molecular structure of DHA was added to the CHARMM force field by “simulation” module, and DHA targets against ESCA were solvated by “solvation” module. The molecular dynamics simulation parameters were set by “standard dynamics cascade module for the targets added with the solvent system. The system temperature was elevated from 50 K to 300 K under 100 ns of analog sampling; the time step was set to 1 ns; and other parameters were set to default values. The Root Mean Square Deviation (RMSD) as well as Root Mean Square Float (RMSF) values were analyzed.

## Results

### Network pharmacology determines dihydroartemisinin targets against esophageal carcinoma

**FIGURE 1:**
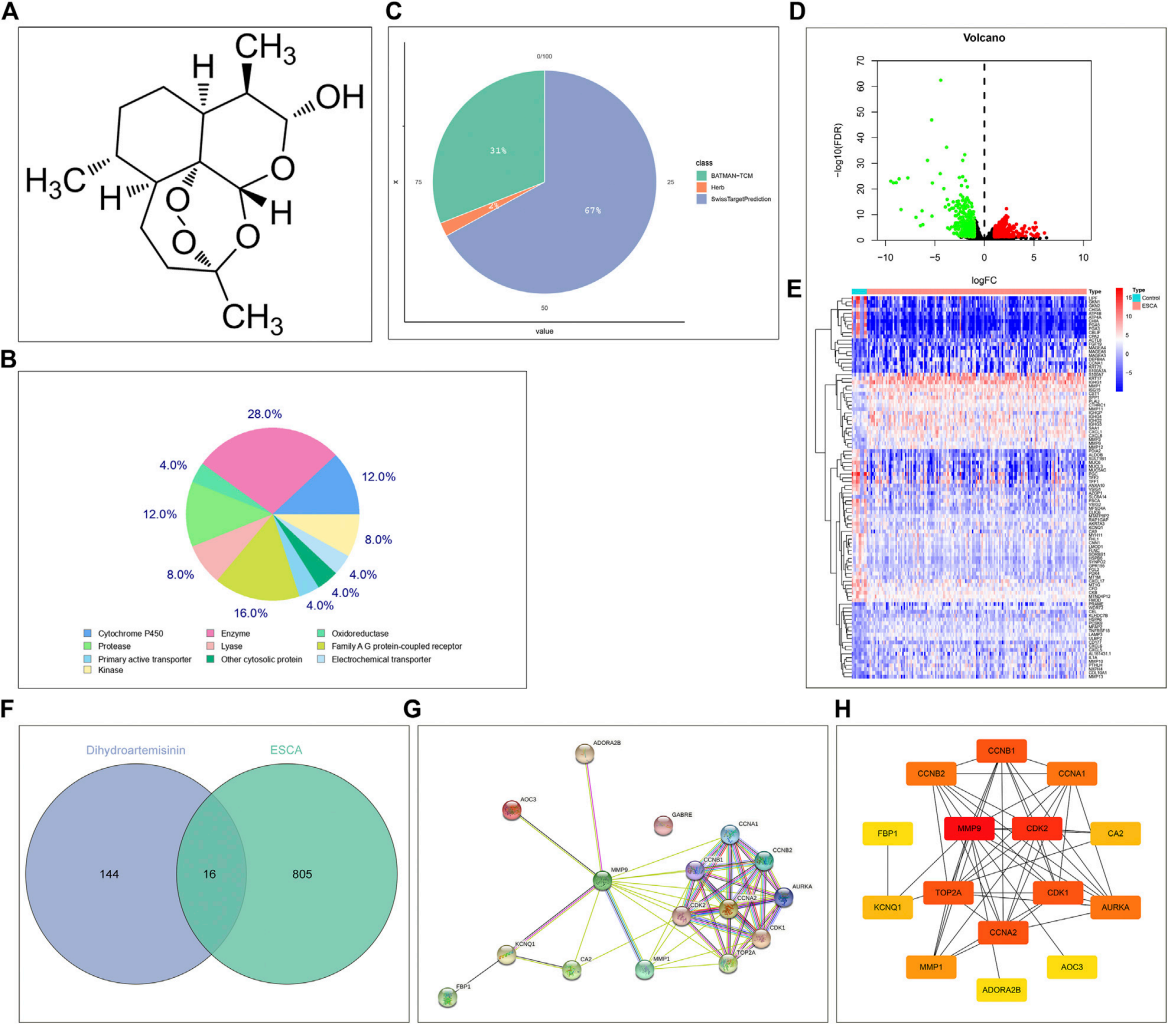
Network pharmacology determines DHA targets against ESCA. **(A)** The 2D chemical structure of DHA from the PubChem chemical information resource. **(B)** The target class distribution for the first 25 DHA targets predicted by the SwissTargetPrediction platform. **(C)** The distribution of DHA targets predicted by the SwissTargetPrediction, HERB, as well as BATMAN-TCM platforms. **(D)** Volcano diagram of the DEGs between 161 ESCA and 11 normal tissue specimens in TCGA-ESCA dataset. Red, up-regulation; green, down-regulation. **(E)** Heatmap of the expression values of the first 100 most significantly DEGs between 161 ESCA and 11 normal tissue specimens in TCGA-ESCA dataset. **(F)** Venn diagram of the shared genes between DHA targets and DEGs. **(G,H)** The PPI network of DHA targets against ESCA through the **(G)** STRING online tool or **(H)** cytoHubba plugin of Cytoscape tool.

From the PubChem chemical information resource, we accessed the 2D chemical structure of DHA, as illustrated in Figure 1A. Through integrating the SwissTargetPrediction, HERB, as well as BATMAN-TCM platforms, potential therapeutic targets of DHA were predicted. In accordance with the SwissTargetPrediction platform, the target class distribution for the first 25 DHA targets was visualized (Figure 1B). Enzyme was the predominant target class, occupying 28.0%. In total, 111, 3, and 51 DHA targets were separately predicted by the SwissTargetPrediction, HERB, as well as BATMAN-TCM platforms (Figure 1C). Under merging and deduplication, 160 DHA targets were finally acquired (Supplementary Table S1).

In TCGA-ESCA dataset, 821 DEGs (comprising 429 genes with up-regulation as well as 392 genes with down-regulation) between 161 ESCA and 11 normal tissue specimens were screened in line with the screening condition of adjusted *p*-value < 0.01 together with |log2 fold-change|>1 (Figure 1D). The specific information of DEGs was listed in Supplementary Table S2. The first 100 most significantly DEGs were depicted in Figure 1E. Venn diagram illustrated 16 DHA targets against ESCA shared by DHA targets and DEGs (Figure 1F), comprising MMP9, CA2, FBP1, CCNA1, CCNA2, CDK2, AOC3, MMP1, TOP2A, AURKA, CDK1, CCNB1, CCNB2, KCNQ1, ADORA2B, and GABRE. Their closely interactions were investigated in the PPI network (Figures 1G,H and Table 1).

**TABLE 1:** DHA targets against ESC ranked by degree.

Gene symbol	Log2 fold-change	Adjusted *p*-value	Degree
MMP9	3.239293	2.35E-05	13
CA2	−2.37148	8.01E-08	3
FBP1	−1.21561	0.00363	1
CCNA1	4.166111	0.004252	8
CCNA2	1.499277	9.59E-08	9
CDK2	1.037182	6.92E-05	10
AOC3	−2.21232	7.01E-09	1
MMP1	2.947104	7.96E-05	6
TOP2A	2.52532	3.19E-08	9
AURKA	1.827825	2.06E-09	8
CDK1	1.828233	1.24E-10	9
CCNB1	1.837112	7.43E-10	9
CCNB2	1.771093	4.36E-10	8
KCNQ1	−3.12088	1.83E-15	3
ADORA2B	1.488384	0.001348	1
GABRE	2.197392	0.006224	1

### Establishment and verification of a reliable prognostic signature for esophageal carcinoma

**FIGURE 2:**
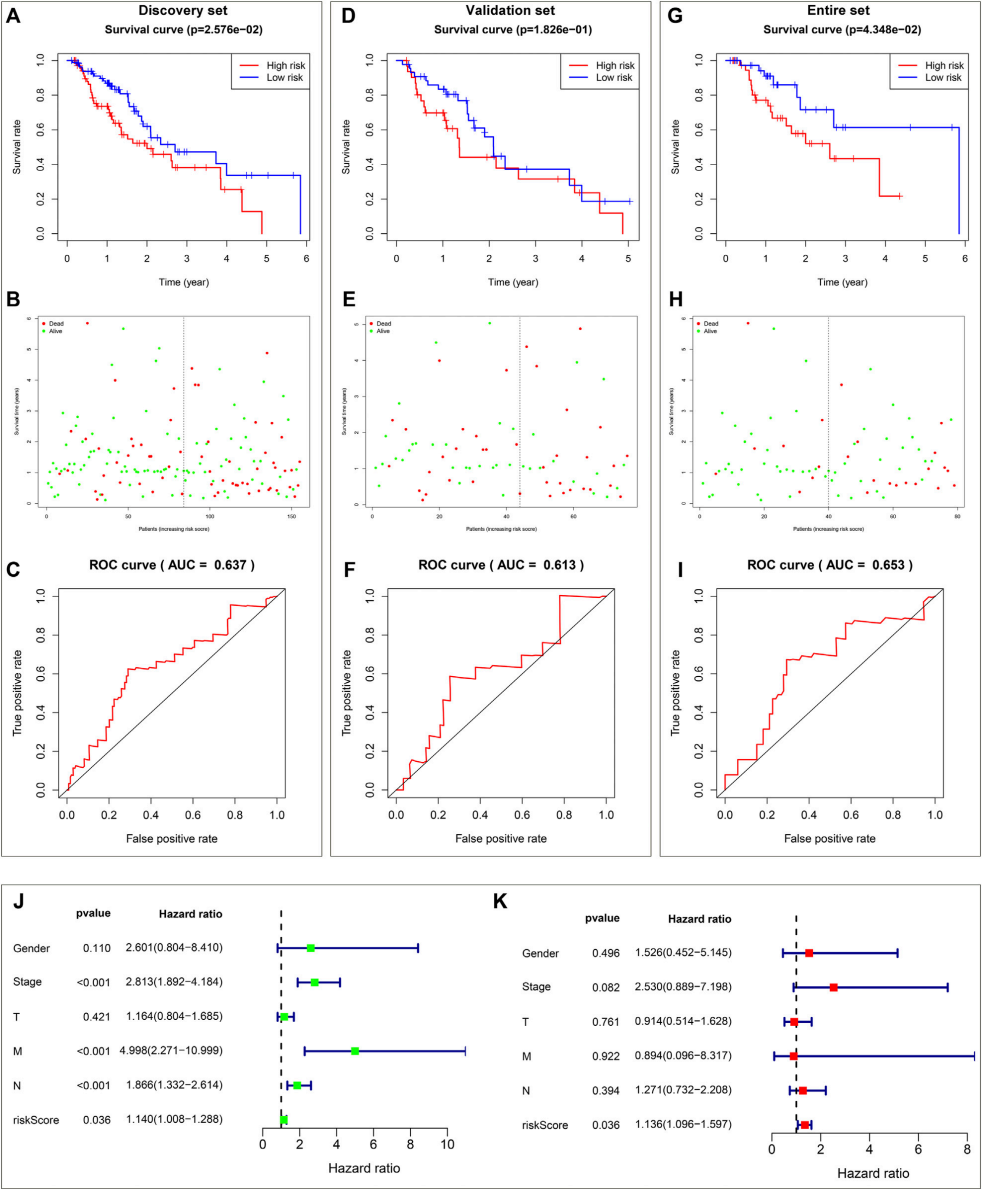
Establishment and verification of a reliable prognostic signature for ESCA cases in TCGA-ESCA dataset. **(A)** Kaplan-Meier OS curves and log-rank test between high- and low-risk subpopulations in discovery set. **(B)** Distribution of alive and dead status in high- and low-risk subpopulations in discovery set. **(C)** ROC curve on the basis of the prognostic signature in discovery set. **(D)** Kaplan-Meier OS curves and log-rank test between high- and low-risk subpopulations in validation set. **(E)** Distribution of alive and dead status in high- and low-risk subpopulations in validation set. **(F)** ROC curve of the prognostic signature in validation set. **(G)** Kaplan-Meier OS curves and log-rank test between high- and low-risk subpopulations in entire set. **(H)** Distribution of alive and dead status in high- and low-risk subpopulations in entire set. **(I)** ROC curve based on the prognostic signature in entire set. **(J)** Forest diagram of univariate cox regression analysis for the associations of clinicopathological factors and the prognostic signature with ESCA cases’ OS. **(K)** Forest diagram of multivariate cox regression analysis for the associations of clinicopathological factors and the prognostic signature with ESCA cases’ OS.

The prognostic implication of DHA targets against ESCA was investigated in TCGA-ESCA dataset. Univariate cox regression analysis demonstrated that AURKA, and ADORA2B significantly correlated to ESCA cases’ OS. AURKA, and ADORA2B were utilized for establishing a multivariate cox regression model. The risk score was computed on the basis of the coefficients and expression values of AURKA, and ADORA2B. TCGA-ESCA cases were equally divided into discovery set and validation set. In discovery set, we assigned cases into high- and low-risk subpopulations. Survival difference between subpopulations was assessed. High-risk subpopulation exhibited worse OS outcome in comparison to low-risk subpopulation (Figure 2A). Survival status was also compared between subpopulations. More dead cases were investigated in high-risk subpopulation (Figure 2B). For evaluating whether the prognostic signature enabled to estimate ESCA cases’ OS, ROC curve was plotted. In Figure 2C, the AUC value was 0.637, indicative of the excellent performance of the prognostic signature in estimating ESCA cases’ OS. The prognostic signature was further verified in validation set and entire set. As expected, it exhibited the remarkable advantage in ESCA cases’ prognosis prediction (Figures 2D–I), indicating the clinical generalizability of this signature.

### The prognostic signature independently predicts esophageal carcinoma patients’ prognosis

Utilizing univariate cox regression analysis, we investigated the associations of clinicopathological factors and the prognostic signature with ESCA cases’ OS. As illustrated in Figure 2J, the prognostic signature along with pathological stage, N stage and M stage were linked to worse ESCA cases’ OS. Multivariate cox regression analysis was conducted for estimating which factors independently predicted ESCA cases’ OS. As a result, the prognostic signature acted as an independent risk factor (Figure 2K).

### Dihydroartemisinin targets against esophageal carcinoma correlate to cell cycle progression

**FIGURE 3:**
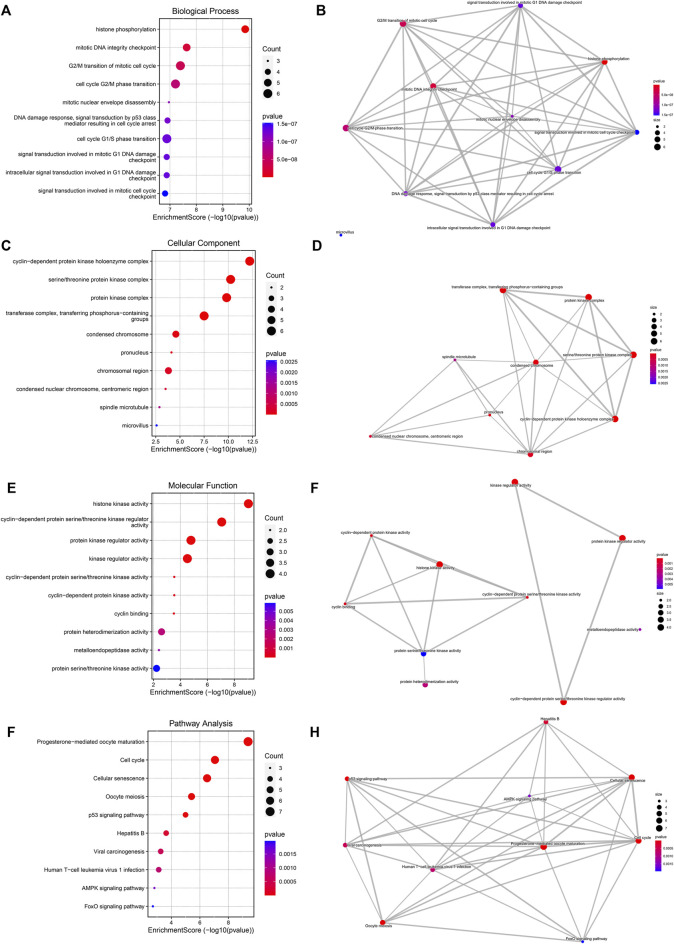
Biological features of DHA targets against ESCA. **(A)** The first ten biological process terms enriched by DHA targets against ESCA. **(B)** Interactions between the first ten biological process terms. **(C)** The first ten cellular component terms enriched by DHA targets against ESCA. **(D)** Interactions between the first ten cellular component terms. **(E)** The first ten molecular function terms enriched by DHA targets against ESCA. **(F)** Interactions between the first ten molecular function terms. **(G)** The first ten KEGG pathways enriched by DHA targets against ESCA. **(H)** Interactions between the first ten KEGG pathways.

Biological features of DHA targets against ESCA were probed out utilizing GO and KEGG enrichment approach. For biological process, histone phosphorylation, mitotic DNA integrity checkpoint, G2/M transition of mitotic cell cycle, cell cycle G2/M phase transition, mitotic nuclear envelope disassembly, DNA damage response, signal transduction by p53 class mediator resulting in cell cycle arrest, cell cycle G1/S phase transition, signal transduction involved in mitotic G1 DNA damage checkpoint, intracellular signal transduction involved in G1 DNA damage checkpoint and signaling transduction involved in mitotic cell cycle checkpoint were significantly linked to DHA targets against ESCA (Figures 3A,B). Cellular components of cyclin-dependent protein kinase holoenzyme complex, serine/threonine protein kinase complex, protein kinase complex, transferase complex, transferring phosphorus-containing groups, condensed chromosome, pronucleus, chromosomal region, condensed nuclear chromosome, centromeric region, spindle microtubule, and microvillus were remarkably enriched by DHA targets against ESCA (Figures 3C,D). Also, they correlated to molecular functions of histone kinase activity, cyclin-dependent serine/threonine kinase regulator activity, protein kinase regulator activity, kinase regulator activity, cyclin-dependent protein serine/threonine kinase activity, cyclin-dependent protein kinase activity, cyclin binding, protein heterodimerization activity, metalloendopeptidase activity as well as protein serine/threonine kinase activity (Figures 3E,F). As illustrated in KEGG pathway enrichment results, progesterone-mediated oocyte maturation, cell cycle, cellular senescence, oocyte meiosis, p53 signaling pathway, hepatitis B, viral carcinogenesis, human T-cell leukemia virus one infection, AMPK signaling pathway as well as FoxO signaling pathway (Figures 3G,H). Altogether, DHA targets against ESCA might participate in mediating cell cycle progression.

### Dihydroartemisinin targets against esophageal carcinoma (AURKA and ADORA2B) correlates to prognosis and pathological stage of esophageal carcinoma

**FIGURE 4:**
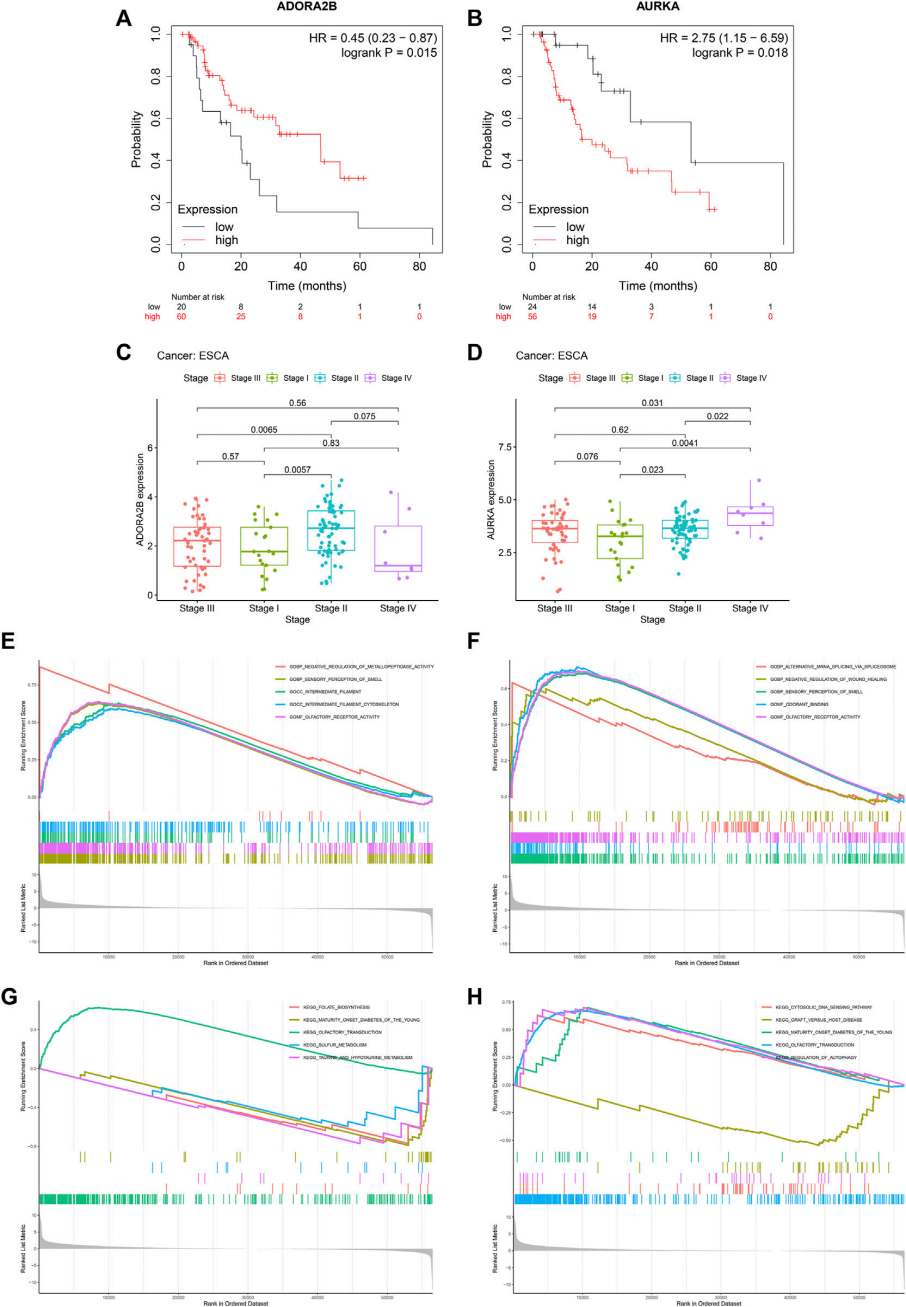
DHA targets against ESCA (ADORA2B and AURKA) correlate to prognosis and pathological stage of ESCA as well as tumorigenic pathways. **(A)** Kaplan-Meier OS curves between high and low ADORA2B expression subpopulations in TCGA-ESCA dataset. **(B)** Kaplan-Meier OS curves between high and low AURKA expression subpopulations in TCGA-ESCA dataset. **(C,D)** ADORA2B and AURKA expression across distinct pathological stages. **(E,F)** GO enrichment results of ADORA2B and AURKA. **(G,H)** KEGG pathway enrichment results of ADORA2B and AURKA.

Among DHA targets against ESCA, DORA2B, and AURKA significantly correlated to ESCA prognosis. Up-regulated ADORA2B was linked to better OS, while up-regulated AURKA was correlated to worse OS in TCGA-ESCA dataset (Figures 4A,B). Further analysis for the associations of ADORA2B and AURKA with pathological stage of ESCA was carried out. In Figure 4C, stage II cases exhibited higher ADORA2B expression. Additionally, AURKA expression displayed positive associations to pathological stage (Figure 4D), indicating that AURKA was linked to ESCA progression.

### Mechanisms underlying ADORA2B and AURKA

Mechanisms underlying ADORA2B and AURKA were further probed out utilizing GSEA. For GO enrichment results, negative regulation of metallopeptidase activity, sensory perception of smell, intermediate filament, intermediate filament cytoskeleton and olfactory receptor activity exhibited positive interactions to ADORA2B (Figure 4E). Meanwhile, alternative mRNA splicing *via* spliceosome, negative regulation of wound healing, sensory perception of smell, odorant binding and olfactory receptor activity displayed positive correlations to AURKA (Figure 4F). For KEGG pathway enrichment results, folate biosynthesis, maturity onset diabetes of the young, olfactory transduction, sulfur metabolism as well as taurine and hypotaurine metabolism were positively linked to ADORA2B (Figure 4G). Also, cytosolic DNA sensing pathway, graft *versus* host disease, maturity onset diabetes of the young, olfactory transduction, and regulation of autophagy exhibited positive correlations to AURKA (Figure 4H).

### Molecular docking between dihydroartemisinin and potential targets

**FIGURE 5:**
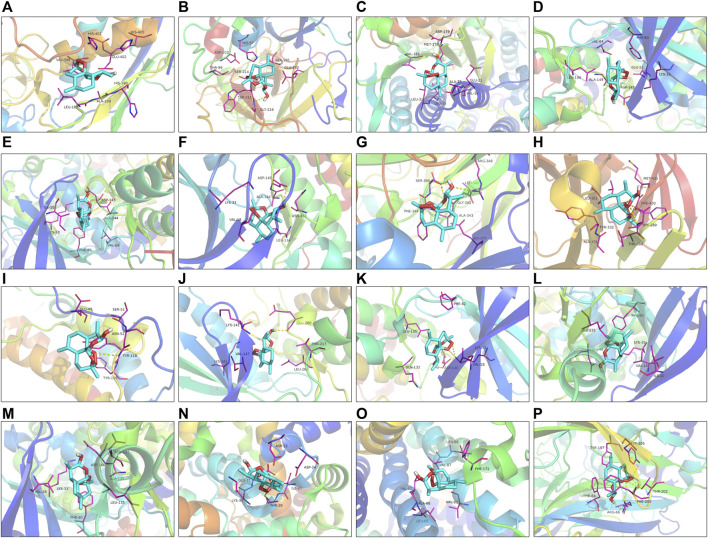
The 3D molecular docking models between DHA and potential targets. **(A)** MMP9; **(B)** CA2; **(C)** FBP1; **(D)** CCNA1; **(E)** CCNA2; **(F)** CDK2; **(G)** AOC3; **(H)** MMP1; **(I)** TOP2A; **(J)** AURKA; **(K)** CDK1; **(L)** CCNB1; **(M)** CCNB2; **(N)** KCNQ1; **(O)** ADORA2B; and **(P)** GABRE.

**FIGURE 6:**
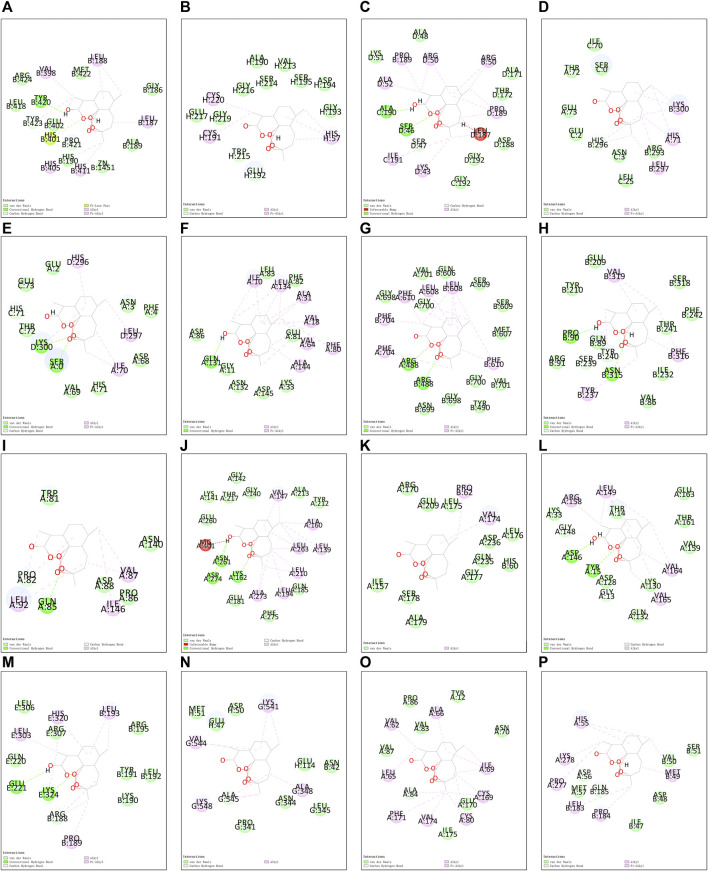
The 2D molecular docking models between DHA and potential targets. **(A)** MMP9; **(B)** CA2; **(C)** FBP1; **(D)** CCNA1; **(E)** CCNA2; **(F)** CDK2; **(G)** AOC3; **(H)** MMP1; **(I)** TOP2A; **(J)** AURKA; **(K)** CDK1; **(L)** CCNB1; **(M)** CCNB2; **(N)** KCNQ1; **(O)** ADORA2B; and **(P)** GABRE.

Utilizing Chem3D, we obtained the 3D structure of DHA with mol*2 format, and downloaded the 3D structure of potential targets from the PDB database with pdb format. Through AutoDockTools 1.5.6 software, the 3D structure of DHA and potential targets were converted to pdbqt format, and thus searched for the active pocket. Afterwards, we run the Vina script to compute the binding energies of DHA and potential targets. As listed in Table 2, the binding energies of the docking bodies formed by DHA targets against ESCA (MMP9, CA2, FBP1, CCNA1, CCNA2, CDK2, AOC3, MMP1, TOP2A, AURKA, CDK1, CCNB1, CCNB2, KCNQ1, ADORA2B, and GABRE) and DHA were all <−5.0 kcal mol^−1^, indicative of the stable docking. Additionally, we computed LibDockScore to dock DHA and its potential targets by use of Discovery Studio 2019 software. As a result, in each potential target, docking site of DHA can be found, and the LibDockScore of the docking model formed by DHA and each target was >50. The 3D and 2D molecular docking models between DHA and potential targets (MMP9, CA2, FBP1, CCNA1, CCNA2, CDK2, AOC3, MMP1, TOP2A, AURKA, CDK1, CCNB1, CCNB2, KCNQ1, ADORA2B, and GABRE) were separately displayed as Figures 5A–P and Figures 6A–P.

**TABLE 2:** Molecular docking between DHA and potential targets.

Domain	Compound	Vina (kcal·mol^−1^)	RMSD	DS(LibDockScore)	Hydrogen bond interaction	Hydrophobic interaction
MMP9(1GKC)	DHA	−7.2	1.221	92.2116	TYR:420,TYR:423,PRO:421	LEU:188,VAL:398,HIS:401,HIS:405,HIS:411,LEU:187
CA2(3F6U)	DHA	−5.9	2.163	76.5684	TRP:215,GLU:192	HIS:57,CYS:220,CYS:191
FBP1(7CVH)	DHA	−6.4	1.678	99.8336	ALA:190,SER:46,SER:47	ARG:50,PRO:189,ALA:52,ILE:191,LYS:43,LEU:187
CCNA1(2G9X)	DHA	−9.0	1.488	80.4867	HIS:296	LEU:297,HIS:71,LYS:300
CCNA2(1OI9)	DHA	−9.6	1.068	74.8124	HIS:71,LYS:300,SER:0	HIS:296,LEU:297,ILE:70
CDK2 (1GIH)	DHA	−9.6	0.978	79.1237	GLN:131	ILE:10,LEU:134,ALA:31,VAL:18,VAL:64,ALA:144,PHE:80
AOC3(2Y74)	DHA	−8.7	1.791	94.64	ARG:488	LEU:608,PHE:610,PHE:704
MMP1(1SU3)	DHA	72.6	1.825	86.2719	PRO:90,SER:239,TYR:240,ASN:315	PHE:316,TYR:237,VAL:319
TOP2A (5NNE)	DHA	−4.0	1.153	66.8962	GLN:85	VAL:87,ILE:146,LEU:92
AURKA (5DOS)	DHA	−8.9	1.397	86.7565	ASN:261,ASP:274,LYS:162	MG:401,ALA:273,LEU:194,LEU:210,LEU:263,LEU:139,ALA:160,VAL:147
CDK1 (6GU6)	DHA	−8.8	1.877	50.3516	-	VAL:174,PRO:62
CCNB1(4Y72)	DHA	−10.4	1.311	92.661	ASP:146,TYR:15,GLY:13	ARG:158,LEU:149,VAL:164,VAL:165
CCNB2(5LQF)	DHA	−9.4	1.340	85.0795	GLU:221,LYS:324,ARG:188,ARG:307	LEU:193,HIS:320,LEU:303,PRO:189
KCNQ1(6B8M)	DHA	7.8	1.842	71.392	ALA:545	LYS:541,VAL:544,LYS:548,ALA:348
ADORA2B (5UEN)	DHA	−8.4	1.811	88.3329	ALA:84	ALA:66,VAL:62,LEU:65,PHE:171,VAL:174,CYS:80,CYS:169,ILE:69
GABRE(6DW0)	DHA	10.6	1.637	80.0045	ASP:56,GLN:185	HIS:55,LYS:278,PRO:277,LEU:183,PRO:184,MET:49

RMSD, root mean square deviation; DHA, dihydroartemisinin.

### Molecular dynamics simulation

**FIGURE 7:**
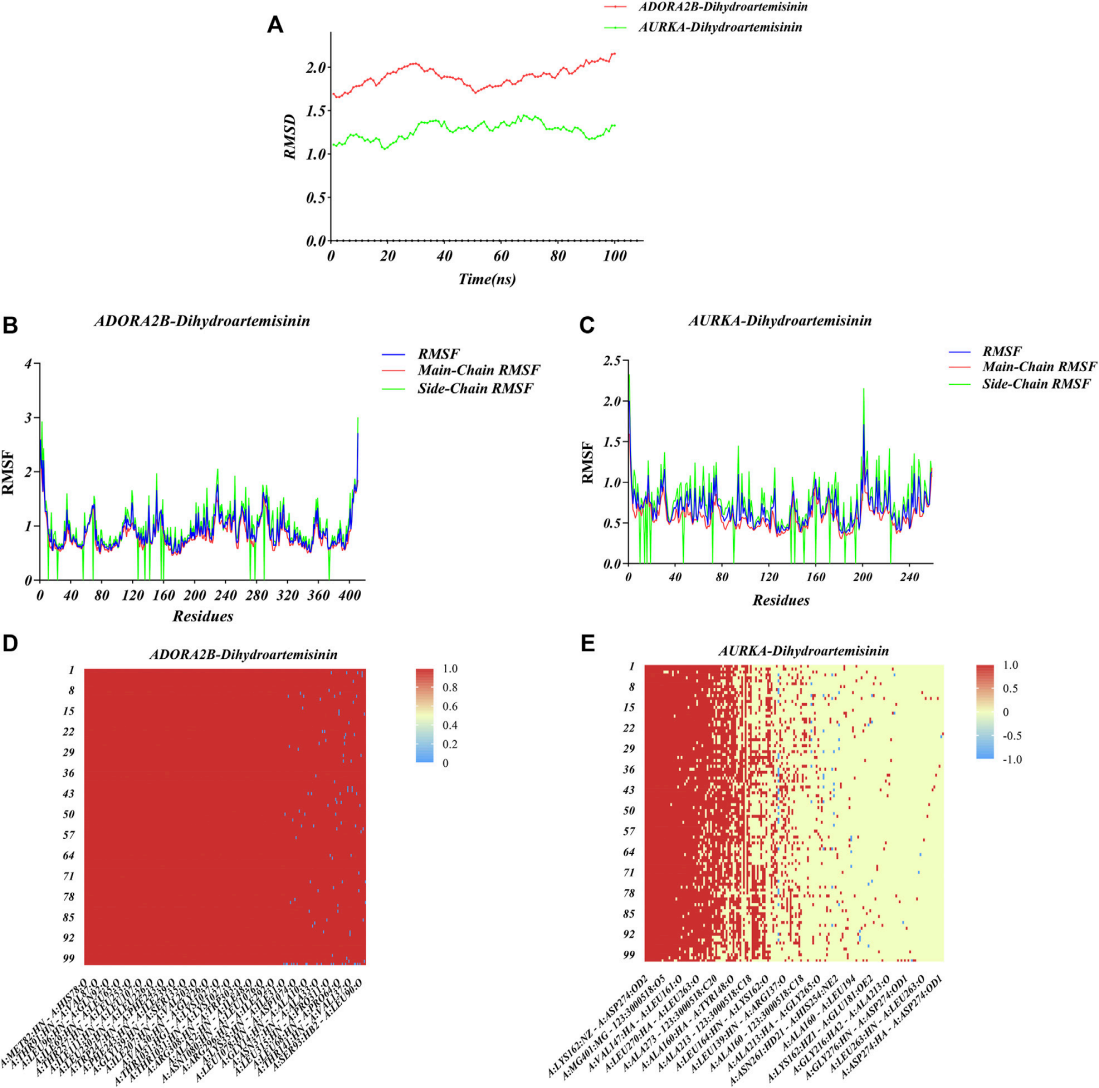
Molecular dynamics simulation of ADORA2B-DHA and AURKA-DHA complexes. **(A)** Alterations in RMSD values during the process of molecular dynamics simulation of ADORA2B-DHA (red) and AURKA-DHA (green) complexes. **(B,C)** Alterations in RMSF values during the process of molecular dynamics simulation of **(B)** ADORA2B-DHA and **(C)** AURKA-DHA complexes. **(D,E)** Heatmaps of hydrogen bonding interactions of **(D)** ADORA2B-DHA and **(E)** AURKA-DHA complexes.

**FIGURE 8:**
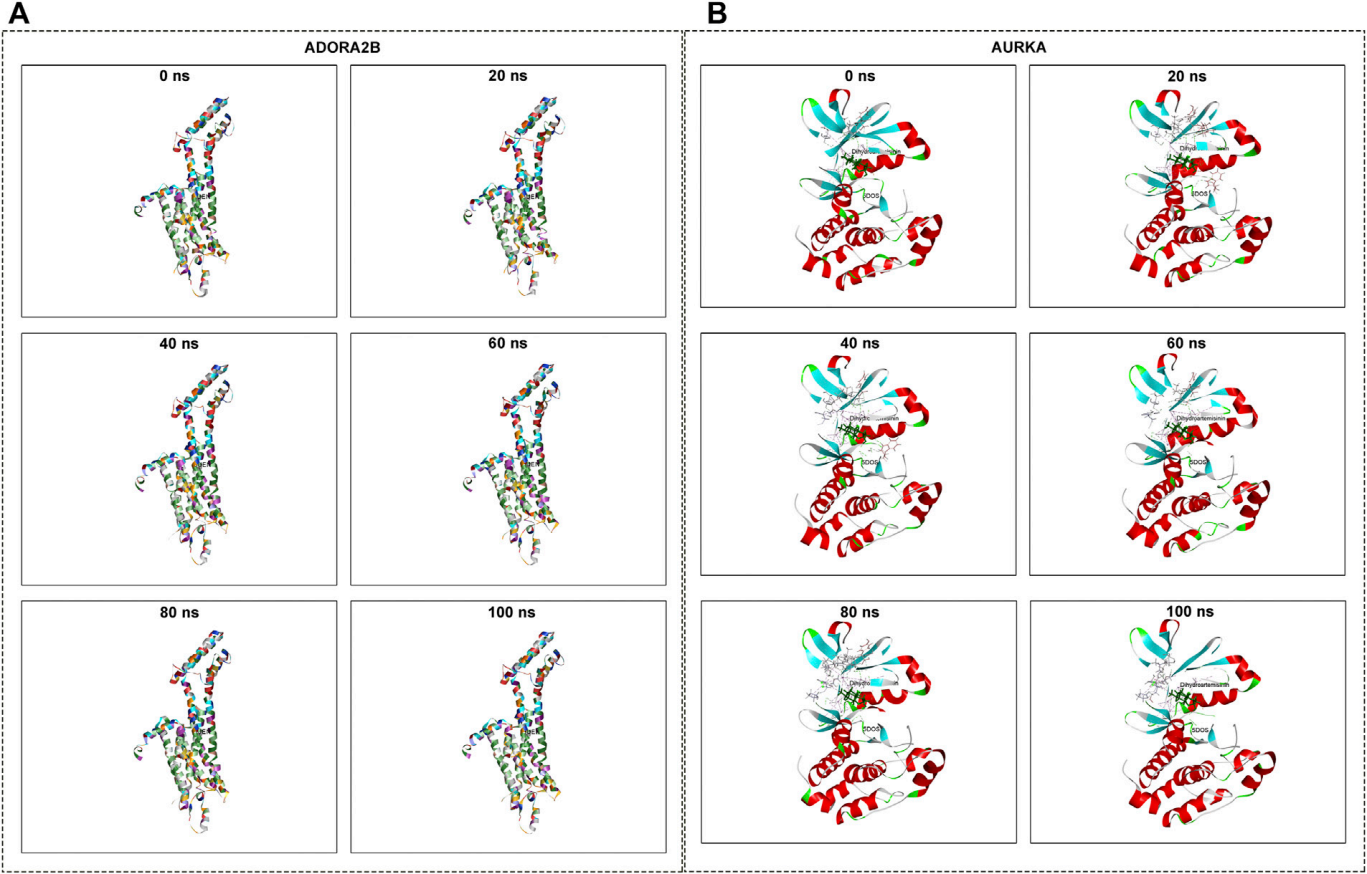
Molecular dynamics simulation of ADORA2B-DHA and AURKA-DHA complexes. **(A)** ADORA2B-DHA complex; **(B)** AURKA-DHA complex.

The conformations of ADORA2B-DHA and AURKADHA molecular docking were used for subsequent molecular dynamics simulation analysis. The ADORA2B-DHA complex was added by 16,738 water molecules, 44 sodium as well as 53 chloride, while the AURKA-DHA complex was added by 5,688 water molecules, 15 sodium as well as 20 chloride. We further investigated the structural stability of ADORA2B-DHA and AURKADHA complexes during molecular dynamics simulation. Meanwhile, the RMSD values of the two complexes were computed during 100 ns molecular dynamics simulation. The ADORA2B-DHA and AURKADHA complexes were stable following 100 ns molecular dynamics simulation, as illustrated in Figure 7A. The RMSD value of ADORA2B-DHA complex mainly fluctuated from 1.65612 to 2.15719, and the mean RMSD value was 1.89383; while the RMSD value of AURKA-DHA complex mainly fluctuated from 1.05842 to 1.44505, and its mean RMSD value was 1.26881 (Figure 7A). The RMSD fluctuation values of the two complexes were all within a reasonable range, demonstrating that the ADORA2B-DHA and AURKA-DHA complexes were in a stable state during the process of molecular dynamics simulation. For analyzing the volatility of distinct amino acids in the two complexes during the process of molecular dynamics simulation, we also computed the RMSF values of all amino acids during the simulation. ADORA2B-DHA complex fluctuated greatly around amino acids Ser6, Ala7, Phe8, Leu315, and Phe316 (Figure 7B); AURKA-DHA complex fluctuated greatly around amino acids Gln127, Trp128, Ala129, Phe329, Glu330, Ala331, and Asn332 (Figure 7C). The heatmaps of hydrogen bonds during the ADORA2B-DHA and AURKADHA molecular docking were separately exhibited in Figures 7D,E. Hydrogen bonding interactions were present in most conformations of ADORA2B-DHA and AURKADHA molecular docking, demonstrating that these hydrogen bonds were persistent and stable. The molecular dynamics simulation results of ADORA2B-DHA and AURKA-DHA complexes were separately displayed in Figures 8A,B.

## Discussion

At present, the paradigms of development of single-targeted therapeutic agents are challenging, primarily because of lack of effectiveness as well as resistance. Therefore, natural compounds selectively acting on two or more targets exhibit higher efficacy in comparison to single-target drugs. The current study employed network pharmacology as well as molecular docking approaches to predict multi-targets and multi-pathways of DHA against ESCA.

Previous studies have proposed that DHA exhibits the anti-ESCA property through targeting distinct molecules as well as pathways (Li et al., 2019; Ma et al., 2020). Thus, we presented a systematic analysis for unveiling DHA targets against ESCA. Through integrating the SwissTargetPrediction, HERB and BATMAN-TCM platforms, 160 DHA targets were finally acquired. Meanwhile, 821 DEGs between 161 ESCA and 11 normal tissue specimens in TCGA-ESCA dataset were screened, which might participate in ESCA initiation as well as progression. Following the intersection of DHA targets and DEGs, we eventually determined 16 DHA targets against ESCA, comprising MMP9, CA2, FBP1, CCNA1, CCNA2, CDK2, AOC3, MMP1, TOP2A, AURKA, CDK1, CCNB1, CCNB2, KCNQ1, ADORA2B, and GABRE. These DHA targets against ESCA remarkably correlated to cell cycle progression. Evidence demonstrates that DHA triggers cell cycle arrest during tumor progression (Lin et al., 2016; Saidi et al., 2021).

Among them, ADORA2B as well as AURKA correlated to ESCA cases’ OS. AURKA is a membrane of serine/threonine kinase family, and its activation is essential for cell division process by modulating mitosis (Du et al., 2021). AURKA accelerates ESCC progression *via* improving the activity of distinct pathways such as EGFR-PI3K-Akt (Du et al., 2020; Shi et al., 2021). Nevertheless, whether ADORA2B participates in ESCA remains uncharted. On the basis of ADORA2B as well as AURKA, a prognostic signature was developed for survival prediction. Despite the reliability and independency of the prognostic signature in ESCA cases’ OS, large prospective ESCA cohorts are required for verifying it.

Molecular docking uses mapping software to place small molecule compounds on the binding region of macromolecular targets, and then calculates parameters to predict the binding ability and binding mode of the two (Tao et al., 2020). Through the strength of the binding ability, the possible mechanisms of action of the small molecule compound can be preliminarily inferred, and the interaction mode between the small molecule compound and the potential target can be quickly and accurately described, which provides a scientific basis for the preparation of derivatives, and thus shortens the drug development cycle and reduces research and development costs. In this study, through use of molecular docking, the stable binding of DHA to 16 potential targets were verified, and thus speculated the main anti-ESCA targets and interactions. Molecular dynamics simulation can simulate the combination of the two in the natural state, and can restore some reaction processes that cannot be detected by current technical means. We employed molecular dynamics simulations for confirming the stability of the binding of DHA to protein targets (ADORA2B and AURKA). Despite this, experiments are essential for confirming ADORA2B-DHA and AURKA-DHA interactions in ESCA.

## Conclusion

Collectively, the current research unveiled that DHA exerted an anti-ESCA effect through targeting multiple targets (especially ADORA2B and AURKA) as well as multiple pathways (especially cell cycle progression) on the basis of network pharmacology and molecular docking approaches, and thus provided the theoretical basis for the pharmacological research of DHA against ESCA.

## Data Availability

The datasets presented in this study can be found in online repositories. The names of the repository/repositories and accession number(s) can be found in the article/Supplementary Material.

## Supplementary material

Supplementary Table S1 DHA targets predicted by the SwissTargetPrediction, HERB, as well as BATMAN-TCM platforms.

Supplementary Table S2 The specific information of 821 DEGs between 161 ESCA and 11 normal tissue specimens in TCGA-ESCA dataset.

## Abbreviations

ESCA, esophageal carcinoma; ESCC, squamous cell carcinoma; EAC, adenocarcinoma; DHA, dihydroartemisinin; TCGA, The Cancer Genome Atlas; PPI, Protein-protein interaction; STRING, Search Tool for the Retrieval of Interacting Genes; OS, overall survival; ROC, receiver operating characteristic; AUC, area under the curve; GO, Gene Ontology; KEGG, Kyoto Encyclopedia of Genes and Genomes; GSEA, Gene set enrichment analysis; RMSD, Root Mean Square Deviation; RMSF, Root Mean Square Float.
